# Prevalence and correlates of using opioids alone among individuals in a residential treatment program in Michigan: implications for overdose mortality prevention

**DOI:** 10.1186/s12954-022-00723-4

**Published:** 2022-12-03

**Authors:** Rachel E. Gicquelais, Becky L. Genberg, Jessica L. Maksut, Amy S. B. Bohnert, Anne C. Fernandez

**Affiliations:** 1 School of Nursing, University of Wisconsin–Madison, 4257 Signe Skott Cooper Hall, 701 Highland Avenue, Madison, WI 53705 USA; 2 Department of Epidemiology, Johns Hopkins Bloomberg School of Public Health, 615 N. Wolfe St, Baltimore, MD 21205 USA; 3 Department of Anesthesiology, University of Michigan, 2800 Plymouth Rd, Ann Arbor, MI 48109 USA; 4 Veterans Affairs Center for Clinical Management Research, 2215 Fuller Rd., Ann Arbor, MI 48105 USA; 5 Department of Psychiatry, University of Michigan, 2800 Plymouth Rd, Ann Arbor, MI 48109 USA

**Keywords:** Opioids, Overdose, Harm reduction, Stigma, Using alone, Polysubstance use

## Abstract

**Background:**

Avoiding use of opioids while alone reduces overdose fatality risk; however, drug use-related stigma may be a barrier to consistently using opioids in the presence of others.

**Methods:**

We described the frequency of using opioids while alone among 241 people reporting daily heroin use or non-prescribed use of opioid analgesic medications (OAMs) in the month before attending a substance use disorder treatment program in the Midwestern USA. We investigated drug use-related stigma as a correlate of using opioids while alone frequently (very often vs. less frequently or never) and examined overdose risk behaviors associated with using opioids while alone frequently, adjusted for sociodemographic and clinical characteristics.

**Results:**

The sample was a median age of 30 years, 34% female, 79% white, and nearly all (91%) had experienced an overdose. Approximately 63% had used OAMs and 70% used heroin while alone very often in the month before treatment. High levels of anticipated stigma were associated with using either opioid while alone very often (adjusted PR: 1.20, 95% CI: 1.04–1.38). Drinking alcohol and taking sedatives within two hours of OAMs very often (vs. less often or never) and using OAMs in a new setting very often (vs. less often or never) were associated with using OAMs while alone very often. Taking sedatives within two hours of using heroin and using heroin in a new setting very often (vs. less often or never) were associated with using heroin while alone very often.

**Conclusion:**

Anticipated stigma, polysubstance use, and use in a new setting were associated with using opioids while alone. These findings highlight a need for enhanced overdose harm reduction options, such as overdose detection services that can initiate an overdose response if needed. Addressing stigmatizing behaviors in communities may reduce anticipated stigma and support engagement and trust in these services.

**Supplementary Information:**

The online version contains supplementary material available at 10.1186/s12954-022-00723-4.

## Introduction

Using drugs while alone, away from bystanders who could potentially respond to an overdose, may contribute to growing overdose mortality, particularly in the context of COVID-19 and growing social isolation [1]. Fatal overdose deaths involving opioids and/or stimulants commonly occur during solitary use. Across studies of fatal overdose events, 39–83% of overdose deaths occurred when an individual was alone at the time of the overdose [2–9]. Overdose reversal through naloxone administration is an effective strategy to decrease deaths associated with opioid-involved overdose. However, naloxone by itself is inadequate to prevent death in the case of solitary drug use, as no bystanders are present to administer naloxone even if it is available [10].

Using drugs while alone is a well-recognized risk factor for fatal overdose, demonstrated by the recommendation to avoid using drugs while alone in public health overdose harm reduction campaigns (e.g., [11]). However, there is limited research into how commonly this behavior occurs or why and when individuals use opioids while alone vs. in the vicinity of others. Existing estimates suggest wide heterogeneity in the prevalence of solitary use, from 7 to 81% depending on the population studied, drugs used, and route of administration [12–25]. To our knowledge, only one prior study summarized the prevalence of solitary use by opioid type, finding that 7% of a sample of male veterans used heroin while alone and 17% reported non-prescribed opioid analgesic medication use while alone [12]. Solitary use was markedly higher among a subgroup of participants who frequently used heroin. Further, prior studies have primarily focused on injecting while alone [13–15, 17, 22–25] and/or recruited participants from harm reduction service venues commonly accessed by those injecting [16, 19–21]. Taken together, a better understanding of how often people use specific types of opioids and their route of administration while they are alone could help inform future harm reduction programming.

People who use drugs cite several reasons for using substances while alone in prior studies. Qualitative research has suggested that self-stigma, anticipated stigma, and discrimination related to substance use, economic concerns (i.e., sharing drugs when with others), and a sense of urgency to ameliorate withdrawal symptoms may drive decision-making around using while alone [26]. It is not yet known how depression, suicidal thinking, self-stigma, and anticipated stigma are quantitatively associated with solitary use, though these are known correlates of overdose and disengagement in substance use treatment [27–31]. One recent study found that the most common reason people used alone related to preferences for convenience and comfort within the setting of use [20]. Other settings, such as public settings (e.g., outdoors), have been associated with overdose, perhaps related to rushed use in unfamiliar or non-private settings due to fear of law enforcement [29]. Methods to garner privacy when in public or shared spaces (e.g., using behind a closed or locked door [26, 32]) may also raise fatality risk as these can preclude bystander access to an overdose victim.

Several additional behaviors increase the likelihood that an overdose occurs or is fatal, regardless of whether someone uses while alone. Polysubstance use may raise the possibility of drug–drug interactions [33–36]. Alcohol and benzodiazepines are of particular concern when mixed with opioids [34]. Further research is needed to characterize other overdose risk behaviors that may co-occur with using while alone.

This study sought to analyze the prevalence and correlates of using opioids while alone among a sample of patients surveyed during residential substance use disorder treatment by evaluating differences in overdose risk behaviors and psychosocial and behavioral characteristics between individuals who reported frequently using opioids while alone relative to those who did not. First, we describe the prevalence of using opioid analgesic medications and heroin while alone with data from a cross-sectional study of 241 people receiving treatment for a substance use disorder who had used opioids daily in the month prior to treatment. Second, we examine other overdose risk behaviors reported by individuals who use while alone frequently, including polysubstance use (e.g., opioids with benzodiazepines or alcohol). Finally, we examine how several psychosocial and behavioral correlates are associated with using while alone, including stigma related to substance use, depression, living situation, type of opioid used, and route of administration. This research will help inform future overdose harm reduction and prevention interventions tailored to people who use opioids while alone and elucidate possible barriers and solutions to adopting public health recommendations to avoid solitary use.

## Methods

### Analytic sample

Data for the present study came from a cross-sectional survey completed by 817 patients in a residential treatment program for any type of substance use disorder in suburban Michigan during 2014–2016. Participants were in treatment at study enrollment, and the dataset used for this analysis was originally collected to assess eligibility for participation in a randomized controlled trial (ClinicalTrials.gov Identifier: NCT02152397). English-speaking patients aged ≥ 18 years were approached by research staff. Interested participants provided informed consent, self-administered a 45-min paper and pencil survey, and received $5 remuneration. The majority of participants in the parent study, like most of those who receive treatment at the study site, were diverted to treatment from jail, prison, or after other types of involvement with the criminal justice system [37].

Inclusion in the present analysis was restricted to 274 participants who reported using opioids daily in the month before treatment or jail. Specifically, these individuals reported either 1) using heroin for ≥ 7 consecutive days in the month before jail or treatment or 2) using opioid analgesic medications for ≥ 7 consecutive days in the month before jail or treatment and having evidence of recent non-prescribed use of opioid analgesic medications (described further below in Measures). After removing individuals missing key measures described below, 241 participants remained for the analysis.

### Measures

Because many participants were diverted to treatment from jail or prison, measures used in this study referred to time periods when participants were last in the community (i.e., before they attended treatment or went to jail or prison).

#### Daily opioid use eligibility criteria

Participants from the parent study were included in this analysis if they met at least one of two criteria pertaining to opioid use. First, they reported “Yes” when asked, “In the month before you entered treatment or jail, have you used heroin at least 7 days in a row?” Alternatively, participants were eligible if they had evidence of recent non-prescribed use of opioid analgesic medications. This was defined as reporting “Yes” when asked, “In the month before you entered treatment or jail, have you used opioid pain medications at least 7 days in a row?” and additionally having a score indicative of moderate or severe opioid misuse based on the Current Opioid Misuse Measure (COMM) [38–40]. For the COMM, participants were asked six questions about how often (range: 0 [Never] to 4 [Very Often]) in the month before entering treatment or jail they: 1) went to someone other than their prescribing physician to get sufficient pain relief from prescription opioids, 2) took prescription opioids differently than prescribed, 3) needed to take prescription opioids belonging to someone else, 4) took more than prescribed, 5) borrowed prescription opioids from someone else, or 6) used prescription opioids to treat symptoms other than pain. Summed responses from these six items were used to classify the severity of misuse (none/mild: score of 0–9 vs. moderate/severe: score of 10–24) based on thresholds described in prior research [40].

#### Using opioids while alone

Participants were asked, “In the month before you entered treatment or jail, how often have you used opioid pain medications when nobody else was around?” and “In the month before you entered treatment or jail, how often have you used heroin when nobody else was around?” Answer choices were “Never,” “Rarely,” “Sometimes,” “Often,” or “Very Often”. Based on the skewed distributions of responses in the sample (76% of participants reported “Very Often” using any opioid while alone and 98% reported using any opioid while alone “Very Often,” “Often,” or “Sometimes”), three binary indicators were created for main analyses: 1) using opioid analgesic medications while alone very often (vs. never, rarely, sometimes, or often), 2) using heroin while alone very often (vs. never, rarely, sometimes, or often), 3) using either opioid while alone very often (vs. never, rarely, sometimes, or often). Additional binary variables were created for sensitivity analyses, including using while alone very often or often (vs. sometimes, rarely, or never), as well as using while alone very often, often, or sometimes (vs. rarely or never).

#### Overdose risk behaviors

Several additional overdose risk behaviors were examined to determine if any were more frequently reported among those using while alone very often. These included frequency of taking prescription sedatives (e.g., Xanax) within two hours of opioid analgesic medications or within two hours of heroin, drinking alcohol within two hours of using opioid analgesic medications or within two hours of using heroin, and using heroin or opioid analgesic medications in a new setting or place in the month before treatment or jail. To be consistent with the binary outcome measures summarizing using while alone, those who endorsed these behaviors very often (vs. never, rarely, sometimes, or often) were examined in main analyses, with separate indicators for opioid analgesic medications, heroin, and either substance. Other binary variables that combined often or sometimes with very often responses were examined in sensitivity analyses.

#### Sociodemographic, psychosocial, and behavioral variables

Several psychosocial and behavioral correlates of using while alone were examined. First, self-stigma and anticipated stigma were measured using two subscales from the validated Substance Abuse Self-Stigma Scale [41]. Self-stigma was from the 8-item self-devaluation stigma subscale (α = 0.82; example question: “I have the thought that a major reason for my problems with substances is my own poor character”). Anticipated stigma was from the 9-item fear of enacted stigma subscale (α = 0.88; example question: “People think I’m worthless if they know about my substance use history”). Participants rated their agreement with each question on a scale of 1 (never or almost never) to 5 (very often). Questions from each subscale are included in Additional file 1: Table S1.

Other correlates included symptoms of major depressive disorder at the time of the survey based on validated cutoffs (score of 10 or above) from the Patient Health Questionnaire-9 [42], predominant living situation in the past 3 months (temporary housing [rooming house or hotel, halfway house, group home, hospital, inpatient treatment center, jail, prison, shelter, or homeless] vs. stable housing [house, apartment, or living with a friend or family member]), current marital status or cohabitation with a significant other (vs. not), route of administration (i.e., snorting drugs in the month before treatment or jail; injecting drugs in the month before treatment or jail), whether the participant had witnessed an overdose in their lifetime, and whether the participant had personally experienced an overdose in their lifetime. Sociodemographic characteristics included self-reported age, gender (male, female, or another gender), race (categorized as white, black or African American, another race [American Indian/Alaska Native, Native Hawaiian or Other Pacific Islander, Asian, or Other race]) and education level (categorized as high school, GED, or greater vs. less than high school education).

### Statistical analysis

First, frequencies of the main outcomes (i.e., using while alone) and other overdose risk behaviors were described. To further contextualize opioid use behaviors in the sample, differences between those reporting daily use of heroin vs. opioid analgesic medications were also described. Associations between overdose risk behaviors with using heroin or opioid analgesic medications while alone very often (vs. often, sometimes, rarely, or never) in the month before treatment or jail were examined using Poisson generalized estimating equation models with robust standard errors to estimate prevalence ratios (PRs) [43]. Using similar statistical methods, associations between psychosocial and behavioral correlates and using any opioid while alone very often (vs. often, sometimes, rarely, or never) were examined. Both unadjusted and adjusted PRs for these associations were examined. Adjusted models included sociodemographic characteristics (age, race/ethnicity, gender) and other variables associated with using opioids while alone in bivariate analyses. Finally, dichotomous variables comparing those using opioids while alone very often or often vs. never, rarely, or sometimes and variables comparing those using opioids while alone very often, often, or sometimes vs. never or rarely were examined in sensitivity analyses to determine whether conclusions differed when the frequency of overdose risk behaviors were grouped differently.

## Results

We included 241 participants who reported using opioid analgesic medications or heroin at least daily in the month prior to treatment or jail. Approximately 79% of participants were white, 34% were female, and the average age was 30 years (Table 1). Approximately 81% graduated high school or obtained a GED and 55% lived in temporary housing in the past three months. The most common type of temporary housing reported was prison or jail (n = 95), and 10 participants reported being homeless or living in a shelter.

**Table 1:** Descriptive characteristics of participants reporting daily non-prescribed OAM use, heroin use, or both

	Total n (%)	OAMs only n (%)	Heroin only n (%)	Heroin and OAMs n (%)	Chi-squared *p*-value
Total	241 (100)	43 (17.8)^a^	62 (25.7)^a^	136 (56.4)^a^	–
Age, median (IQR)	30 (25–37)	34 (27–46)	29 (25–35)	29 (25–35)	0.03^b^
Female gender	83 (34.4)	11 (25.6)	23 (37.1)	49 (36.0)	0.4
Race					0.2^b^
*African American*	19 (7.9)	6 (14.0)	3 (4.8)	10 (7.4)	
*White*	190 (78.8)	33 (76.7)	46 (74.2)	111 (81.6)	
*Other*	32 (13.3)	4 (9.3)	13 (21.0)	15 (11.0)	
Temporary housing in Past 3 Months	132 (55.2)	22 (51.2)	31 (50.0)	79 (59.0)	0.4
Married or living with someone	43 (17.8)	12 (27.9)	11 (17.7)	20 (14.7)	0.1
High School, GED, or greater education	195 (80.9)	35 (81.4)	56 (90.3)	104 (76.5)	0.07
Symptoms of major depressive disorder	142 (58.9)	25 (58.1)	32 (51.6)	85 (62.5)	0.4
Injected or snorted any drug^d^	229 (95.8)	35 (81.4)	61 (98.3)	133 (99.3)	< 0.001^c^
Snorted any drug very often^d^	50 (20.7)	12 (27.9)	3 (4.8)	35 (25.7)	0.001
Injected any drug very often^d^	152 (63.1)	4 (9.3)	46 (74.2)	102 (75.0)	< 0.001
Experienced an overdose in lifetime	218 (90.5)	38 (88.4)	54 (87.1)	126 (92.6)	0.4^c^
Experienced an overdose in the past year	166 (68.8)	26 (60.5)	36 (58.1)	104 (76.5)	0.01
Witnessed an overdose in lifetime	216 (89.6)	37 (86.0)	56 (90.3)	123 (90.4)	0.7^c^

^a^Percents among total n = 241. ^b^Kruskal–Wallis test. ^c^Fisher’s exact test. ^d^In the month before treatment or jail. Abbreviations: IQR: interquartile range; OAM: opioid analgesic medication

Over half of participants (n = 136, 56%) reported daily use of both opioid analgesic medications and heroin in the month before treatment or jail. Of the remaining participants, 43 (18%) reported daily non-prescribed opioid analgesic medication use but not heroin, and 62 (26%) used heroin daily but not opioid analgesic medications. There were several differences between participants who reported non-prescribed opioid analgesic medication use, heroin use, or both substances. Those who reported non-prescribed opioid analgesic medication use but who had not used heroin tended to be older (median age: 34 years) than either group that used heroin (median age: 29, p = 0.03). Route of administration also differed: > 98% of participants who had used heroin reported injecting or snorting drugs in the month prior to treatment or jail, but only 81% of those misusing opioid analgesic medications reported injecting or snorting (p < 0.001). Personally experiencing an overdose in the past year was reported by 77% of the group who reported both non-prescribed opioid analgesic medication use and heroin use compared to approximately 60% of participants who had used one type of opioid (p = 0.01).

### Prevalence of overdose risk behaviors

**Fig. 1:**
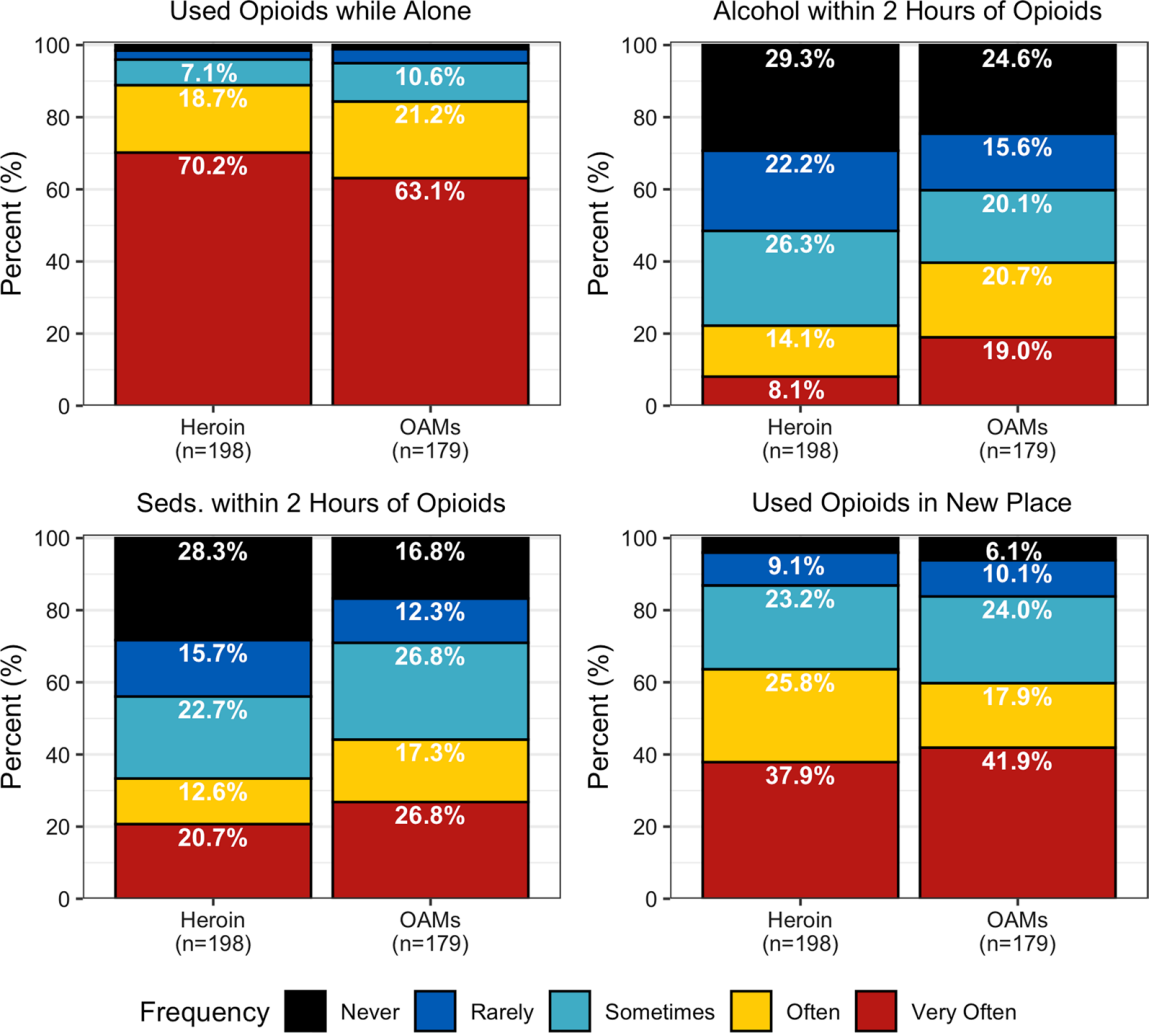
Past-month frequency of overdose risk behaviors among a sample of people using opioids daily, 2014–2016

Among 179 participants who had reported non-prescribed opioid analgesic medication use, 63% reported using them while alone very often in the month before treatment or jail (Fig. 1). Only 1% (n = 2 participants) reported never using while alone, and only 4% (n = 7) rarely used while alone. Among 198 participants who had used heroin, 70% reported using heroin while alone very often in the month before treatment or jail. Only 2% (n = 3) reported never using heroin while alone and 3% (n = 5) reported rarely using heroin while alone.

Among other overdose risk behaviors examined, concomitant opioid analgesic medication use with sedatives or drinking alcohol were more common than concomitant sedative use or drinking with heroin. Whereas 22% of participants reported that they often or very often drank alcohol within 2 h of using heroin, 40% reported often or very often drinking alcohol within 2 h of taking opioid analgesic medications. Similarly, whereas 33% often or very often used sedatives within 2 h of heroin, 44% often or very often used sedatives within 2 h of opioid analgesic medications. Finally, 60% of participants reported using opioids in a new setting or place often or very often, regardless of the type of opioid.

### Associations of overdose risk behaviors with using opioid analgesic medications while alone

**Fig. 2:**
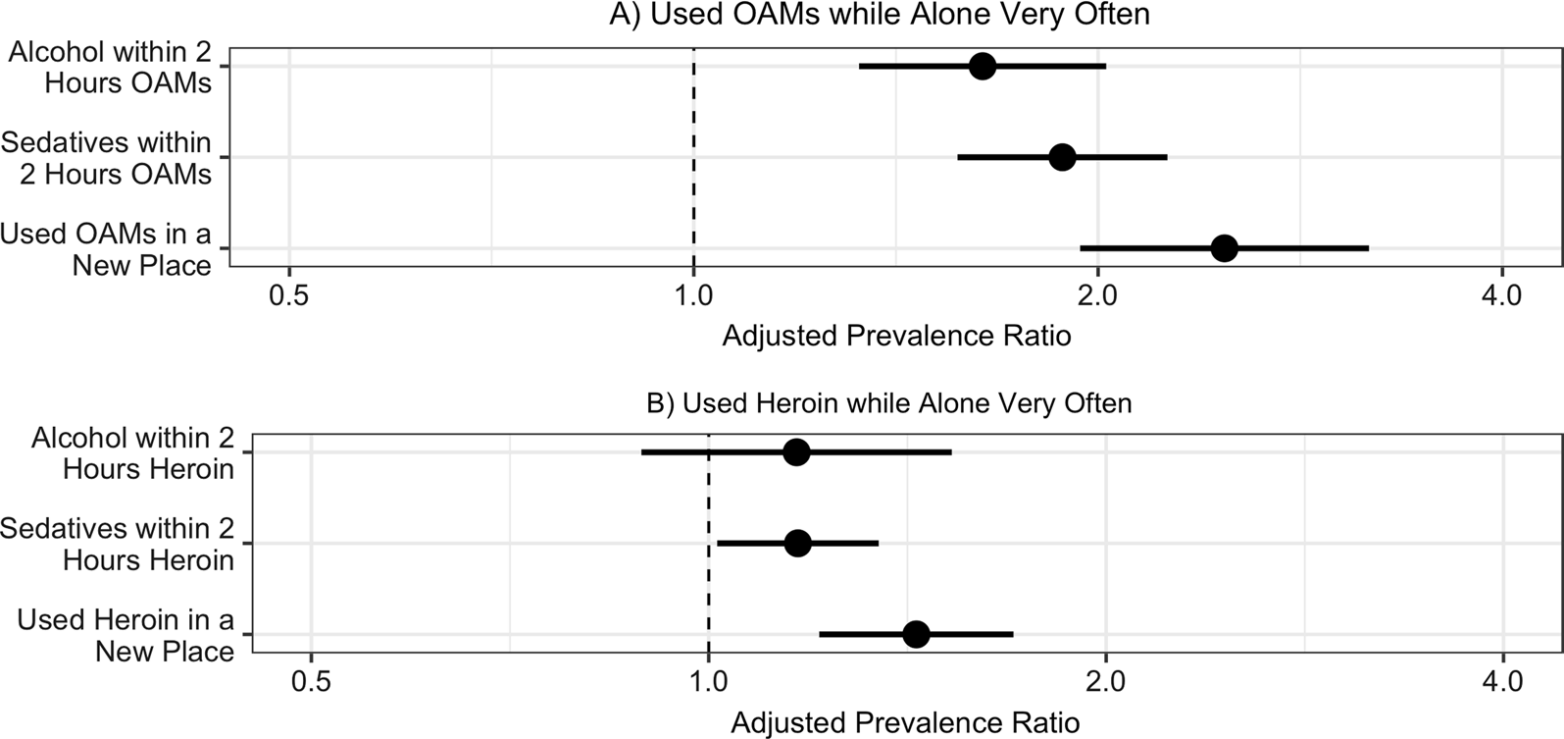
Overdose risk behaviors associated with using opioids while alone very often^a^. Adjusted associations of engaging in several overdose risk behaviors very often (vs. less often or not at all) with using opioid analgesic medications (OAMs, panel A) and heroin (panel B) very often (vs. often, sometimes, rarely, or never). All overdose risk behaviors, including using while alone, were assessed in the past month. After adjustment for age, race, gender, and injecting or snorting any drug, participants who used alcohol or sedatives within two hours of OAMs very often and who used OAMs in a place they didn’t usually use them very often were more likely to also report using OAMs alone very often. After adjustment for age, race, gender, injecting or snorting, and ever experiencing an overdose, using sedatives or OAMs within two hours of using heroin very often and using heroin in a place they didn’t usually use heroin very often were more likely to also report using heroin alone very often. Abbreviations: OAMs: opioid analgesic medications. ^a^Outcome modeled is those who used opioids Very Often vs. Often, Sometimes, Rarely, or Never

In bivariate (Additional file 1: Table S2) and adjusted analysis (Fig. 2), all three overdose risk behaviors examined were associated with using opioid analgesic medications while alone. Participants who reported drinking alcohol within two hours of using opioid analgesic medications very often (vs. often, sometimes, rarely, or never) were 60% more likely (aPR: 1.6, 95% CI: 1.3–2.0) to report using opioid analgesic medications while alone very often after adjustment for sociodemographic characteristics and route of administration. Likewise, participants taking sedatives within two hours of opioid analgesic medications very often were 88% more likely (aPR: 1.9, 95% CI: 1.6–2.3) to report using opioid analgesic medications while alone very often. Those who very often used opioid analgesic medications in a place they did not usually use them were 2.5-fold more likely (aPR: 2.5, 95% CI: 1.9–3.2) to report using opioid analgesic medications while alone very often. In a sensitivity analysis including those who “often” endorsed these behaviors with those who “very often” endorsed them, we found that these associations were attenuated, but similar in direction, suggesting that participants who endorsed using sedatives or using in a new place often or very often were also more likely to report using while alone often or very often (Additional file 1: Table S3). A sensitivity analysis including those who “sometimes” endorsed these behaviors with those who “often” or “very often” endorsed them resulted in further attenuated associations.

### Associations of overdose risk behaviors with using heroin while alone

Two of the three overdose risk behaviors examined were associated with using heroin while alone in bivariate (Additional file 1: Table S4) and adjusted analysis (Fig. 2). Participants who took sedatives within two hours of using heroin very often were 17% more likely (aPR: 1.2, 95% CI: 1.0–1.4) to also report using heroin while alone very often after adjustment for age, race, gender, route of administration, and ever experiencing an overdose. Further, those who reported using heroin in a new setting very often were 44% more likely (aPR: 1.4, 95% CI: 1.2–1.7) to also use heroin while alone very often. Drinking alcohol within two hours of taking heroin was reported by fewer than 10% of participants who reported daily heroin use and was not associated with using while alone.

In sensitivity analyses, using sedatives often or very often (vs. sometimes, rarely, or never) was not associated with using heroin while alone often or very often (aPR: 1.0, 95% CI: 0.9–1.1, Additional file 1: Table S5). The association of using heroin in a new place often or very often (vs. sometimes, rarely, or never) with using while alone often or very often was attenuated by comparison to the main analysis (aPR: 1.2, 95% CI: 1.1–1.4). A sensitivity analysis including those who “sometimes” endorsed these behaviors with those who “often” or “very often” endorsed them resulted in similar associations.

### Psychosocial and behavioral correlates of using any opioid while alone

In adjusted analysis, anticipated stigma and route of administration were associated with using heroin or opioid analgesic medications while alone very often (Table 2). Specifically, participants who scored in the top quartile on the fear of enacted stigma sub-scale (vs. those scoring < 75th percentile) were 20% more likely (aPR: 1.2, 95% CI: 1.0–1.4) to report using any opioid while alone very often after adjustment for sociodemographic characteristics, type of opioid used, route of administration, personal overdose experience, and self-stigma. Further, participants who reported injecting or snorting drugs very often in the month before treatment were 71% more likely (aPR: 1.7, 95% CI: 1.3–2.3) to report using opioids while alone very often. Other correlates we examined were not associated with using any opioid while alone very often in bivariate analyses, including symptoms of major depressive disorder, self-stigma score, predominantly living in temporary housing, or being married or co-habitating with a significant other.

**Table 2:** Psychosocial and behavioral correlates of using any opioid while alone very often

Covariate	Used opioids alone less frequently n (%)	Used opioids alone very often n (%)	Bivariate PR (95% CI)	Adjusted PR (95% CI)
Total	59 (24.5)^a^	182 (75.5)^a^	–	–
Age, median (IQR)	31 (27–45)	30 (25–36)	0.99 (0.98–1.00)	1.00 (0.99–1.01)
Female gender	18 (30.5)	65 (35.7)	1.06 (0.91–1.22)	1.09 (0.95–1.25)
Race				
African American	7 (11.9)	12 (6.6)	ref	ref
White	46 (78.0)	144 (79.1)	1.20 (0.84–1.71)	1.08 (0.77–1.52)
Other	6 (10.2)	26 (14.3)	1.29 (0.88–1.88)	1.10 (0.77–1.56)
Temporary housing in past 3 Mos	28 (47.5)	105 (57.7)	1.11 (0.95–1.28)	–
Married or living with someone	13 (22.0)	30 (16.5)	0.91 (0.74–1.12)	–
High school, GED, or greater education	49 (83.1)	146 (80.2)	0.96 (0.80–1.14)	–
Symptoms of major depressive disorder	36 (61.0)	106 (58.2)	0.97 (0.84–1.12)	–
Heroin and OAM use, mo. before treat. or jail				
Used OAMs (No Heroin) ≥ 7 consecutive days	16 (27.1)	27 (14.8)	Ref	Ref
Used Heroin (No OAMs) ≥ 7 consecutive days	21 (35.6)	41 (22.5)	1.05 (0.79–1.41)	0.83 (0.61–1.12)
Used Heroin & OAMs ≥ 7 consecutive days	22 (37.3)	114 (62.6)	1.33 (1.05–1.70)	0.99 (0.75–1.31)
Injected or snorted any drug very often, mo. before treat. or jail	26 (44.1)	151 (83.0)	1.76 (1.36–2.28)	1.71 (1.30–2.26)
Experienced an overdose in lifetime	48 (81.4)	170 (93.4)	1.49 (1.00–2.22)	1.25 (0.89–1.76)
Experienced an overdose in the past year	35 (59.3)	131 (72.0)	1.16 (0.98–1.38)	–
Witnessed an overdose in lifetime	53 (89.8)	163 (89.6)	0.99 (0.79–1.25)	–
Self-stigma (self-devaluation Z-score), mean (SD)	− 0.02 (0.86)	0.01 (1.04)	1.01 (0.94–1.08)	–
Self-stigma (self-devaluation score top quartile)^b^	8 (13.6)	43 (23.6)	1.15 (1.00–1.33)	0.94 (0.80–1.09)
Anticipated stigma (fear of enacted stigma Z-score), mean (SD)	− 0.20 (0.94)	0.06 (1.01)	1.07 (0.99–1.15)	–
Anticipated stigma (fear of enacted stigma score top quartile)^c^	6 (10.2)	52 (28.6)	1.26 (1.11–1.43)	1.20 (1.04–1.38)

IQR: interquartile range; Mo: month; OAM: opioid analgesic medication; PR: prevalence ratio; SD: standard deviation; Treat: treatment

Outcome modeled is those who used opioids Very Often vs. Often, Sometimes, Rarely, or Never

^a^Percent among total n = 241

^b^Top quartile: ≥ 35 points

^c^Top quartile: ≥ 39 points

In sensitivity analyses (Additional file 1: Table S6), anticipated stigma was not associated with using any opioid while alone often or very often (aPR: 1.1, 95% CI: 1.0–1.2), nor was injecting or snorting drugs often or very often (aPR: 1.2, 95% CI: 1.0–1.5). A sensitivity analysis analyzing predictors of using any opioid while alone sometimes, often, or very often was not possible, as only four participants reporting using both heroin and opioid analgesic medications while alone “never” or “rarely.”

## Discussion

Frequently using opioids while alone was extremely common in this sample, as three-quarters of participants reported using heroin or opioid analgesic medications while alone very often. Using while alone increases the risk of fatal overdose, as solitary use precludes the possibility of a bystander response or call for emergency medical assistance. Participants who used while alone very frequently in our study were also more likely to endorse other overdose risk behaviors compared to those who used while alone less frequently. For example, concomitantly using opioids and sedatives was associated with using heroin or opioid analgesics while alone, a behavior that increases the likelihood that an overdose will occur due to drug–drug interactions [34–36]. We also found that anticipated stigma related to substance use was associated with frequently using while alone.

These results extend previous research. Estimates of the prevalence of using opioids while alone vary widely, from 7 to 81%, depending on the studied population [12–25]. The frequency of using while alone in our study was generally higher than prior research suggests. Most prior studies involved people who injected drugs and those recruited from syringe services or harm reduction programs primarily located in urban areas. Among those examining injecting while alone, 15% to 46% injected while alone at frequencies such as “always,” “all the time,” or “usually” [14, 21–23, 25]. To our knowledge, only one prior study evaluated how commonly people using drugs by any route of administration reported using opioids while alone. One study of Veterans who had recently used opioids (only 7% of whom used by injection at least once in the past month) found that 7% of participants used heroin while alone on at least half the days in the past month, and 17% took a higher dose of prescription opioids than advised while they were alone on at least half the days in the past month [12]. In our study, the vast majority of participants used while alone “very often,” including those using opioid analgesic medications and those who reported other overdose risk behaviors that could further raise the risk of overdose. These findings suggest that interventions aimed at reducing overdose harms related to solitary use might additionally be delivered beyond harm reduction settings, such as in substance use disorder treatment, pain clinics, emergency medical settings, or primary care. These settings could have greater reach to those engaging in non-prescribed use of opioid analgesic medications or located in rural or suburban communities that may have less access to harm reduction services. Using while alone and overdose have only become more prevalent during COVID-19 [13, 44], further emphasizing the potential need to reach more individuals.

One promising new tool that could reach those receiving services across these settings is overdose detection technologies, such as the Never Use Alone telephone hotline [45] and the Brave smartphone application [46]. These technologies set up virtual safer use spaces, similar to brick-and-mortar overdose prevention centers, which are just beginning to emerge and operate in a minimal number of places in the USA [47, 48]. These technologies connect callers about to use drugs with an operator who monitors and facilitates an emergency response if a caller becomes unresponsive. Our finding that using in a new setting was associated with using while alone very often further emphasizes the need for flexible and accessible overdose detection services, a need that telephone hotlines and smartphone applications may be capable of meeting. Rural or other communities where population dispersion makes sustaining substance use disorder treatment and harm reduction programs challenging may also be particularly well-served by these flexible overdose detection technologies.

This study extends a small number of prior qualitative studies [26, 49] supporting that anticipated stigma related to substance use is associated with frequently using opioids while alone. Anticipated stigma is the expectation that one will be rejected, mistreated, or devalued in the future if their identity as a person who uses drugs is found out [31, 50]. Though few studies have focused on stigma and using while alone specifically, prior research exploring reasons underlying preferences to maintain privacy while using drugs may help to explain this relationship. These include distrust of peers or police, the need to be discrete and use quickly to avoid being interrupted or found out, fear of violence or exploitation, and emotional pain [26, 32, 49, 51–55]. These reasons further codify the fundamental role stigma plays in raising overdose risk [31, 56]. Beyond stigma, many people who use drugs while alone do so for highly practical reasons, such as the inability to afford sharing drugs or a preference for using in a convenient and comfortable space [20, 26, 51]. Encouragingly, both of the aforementioned overdose detection technologies we suggest above as a flexible intervention to reduce harms among those engaging in solitary use emphasize a non-stigmatizing environment involving acceptance and respect for privacy in recognition of stigma and fear of punishment as barriers to safer drug use. For example, Never Use Alone’s tagline is, “No judgement, no shaming, no preaching, just love!”.

We also found that polysubstance use was associated with using opioids while alone, further increasing risk of fatal and non-fatal overdose [33–36]. Specifically, using opioids and sedatives (e.g., Xanax) within two hours of one another very often was associated with using opioid analgesic medications or heroin while alone very often, and drinking within two hours of taking opioid analgesic medications very often was associated with using opioid analgesic medications while alone very often. These associations were generally stronger in magnitude among those using opioid analgesic medications than those using heroin, suggesting that overdose harm reduction messaging may not adequately reach those engaging in non-medical use of opioid analgesic medications. Education efforts to reduce or stagger polysubstance use and publicizing overdose detection services beyond harm reduction venues may thus assist in reducing risk among these individuals. Professionals working in settings frequented by people engaging in non-medical use of opioid analgesic medications, such as pharmacies, emergency medical settings, primary care, substance use disorder treatment settings, and pain clinics would further be well-positioned to engage clients in discussions about overdose risk and provide tools like naloxone that reduce harm. Outreach to individuals who recently survived an overdose through community-based mobile response teams offer additional opportunities to deliver harm reduction tools to these individuals and their peer networks as well as connect them with services like substance use treatment [57, 58]. Given the recent uptick in fentanyl contamination of illicitly manufactured pills sold as opioid analgesic medications, this is essential now more than ever [59, 60].

Our study has several limitations. First, the data were collected during 2014–2016, just as illicitly manufactured fentanyl began contaminating the US drug supply, and prior to the COVID-19 pandemic. A recent study suggests the potential that using fentanyl may be associated with using while alone [16], highlighting the importance of this study and further research on this topic. Our study was also a cross-sectional, secondary analysis of people sampled while receiving treatment for a substance use disorder. Thus, we cannot rule out reverse causation (e.g., stigma could result in using alone or vice versa) or assume that the results are generalizable to other populations, though we provide detailed data on substances used in the sample to assist with comparisons to other samples. We additionally aimed to define a sample who used opioids frequently so that using while alone “never” was not conflated with not using opioids at all. However, we were limited in the questions available to define the sample in this secondary analysis. Subsequent research designed to examine using while alone specifically will be able to more rigorously define who is at risk of overdose while alone. We also report on associations between reported overdose risk behaviors (e.g., drinking while taking opioids and using opioids while alone) but cannot comment on whether these behaviors truly co-occurred in the same drug using episode, which would only be possible with detailed, event-level data collected by techniques like ecological momentary assessment. Our results were further sensitive to the frequency threshold (i.e., very often, often, or sometimes) used to create indicators of using while alone and other overdose risk behaviors. Further study of the frequency of these behaviors is needed. Finally, we did not collect and thus could not analyze several key covariates, including those related to motivations for using alone (e.g., economic concerns, withdrawal, suicidal ideation, convenience) or other key factors such as whether the participant resided in a rural versus urban location.

## Conclusions

Recently using opioids while alone was highly common in this sample of people with a history of daily opioid use. Endorsing other overdose risk behaviors very often, such as using sedatives within two hours of opioids, was associated with using opioids while alone very often. Anticipated stigma may also be associated with using opioids while alone. These findings highlight the need for enhanced overdose harm reduction options for people engaging in solitary opioid use, including those using heroin or non-prescribed opioid analgesic medications. This need may be met by broadening the use of novel overdose detection options, such as the Never Use Alone hotline or Brave smartphone application.

## Supplementary Information

**Additional file 1.** **Supplemental Tables S1** (questions from the Substance Abuse Self-Stigma Scale used in the study) and **S2–S6** (supplemental results from sensitivity analyses).

## Data Availability

The datasets used and analyzed during the current study are available from the corresponding author on reasonable request.

## Abbreviations

aPR: Adjusted prevalence ratio
CI: Confidence interval
COMM: Current opioid misuse measure
