# Supplementary material for: Prevalence and correlates of using opioids alone among individuals in a residential treatment program in Michigan: implications for overdose mortality prevention
Source: Harm Reduct J. 2022 Dec 3;19:135. doi: 10.1186/s12954-022-00723-4 (PMC9719663; doi:10.1186/s12954-022-00723-4)
Supplement: Supplementary file 1 — Additional file 1. Supplemental Tables S1 (questions from the Substance Abuse Self-Stigma Scale used in the study) and S2–S6 (supplemental results from sensitivity analyses). [file 12954_2022_723_MOESM1_ESM.docx]

**Supplemental Table 1.** Substance Abuse Self-Stigma Scale Questions from Luoma et al.

| **Stigma Subscale** | **Question** |
| --- | --- |
| Self-devaluation | I have the thought that a major reason for my problems with substances is my own personal flaws. |
|  | I have the thought that I should be ashamed of myself. |
|  | I have the thought that I deserve the bad things that have happened to me. |
|  | I have the thought that I can’t be trusted. |
|  | I feel inferior to people who have never had a problem with substances. |
|  | I feel out of place in the world because of my problems with substances. |
|  | I have the thought that I’ve permanently screwed up my life by using drugs. |
|  | I feel ashamed of myself. |
| Fear of enacted stigma | People think that I’m worthless if they know about my substance use history. |
|  | People around me will always suspect I have returned to using substances. |
|  | People without a substance use history could never really understand me. |
|  | A job interviewer wouldn’t hire me if I mentioned my substance history in a job interview. |
|  | If someone were to find out about my history of substance use, they would expect me to be weak. |
|  | People would be scared of me if they knew about my substance abuse history. |
|  | If someone were to find out about my substance use, they would doubt me. |
|  | People will think I have little talent or skill if they know about my substance history. |
|  | People think the bad things that have happened to me are my fault. |

**Supplemental Table 2. Correlates of Using OAMs while Alone Very Often^a^**

| **Covariate** | **Used OAMs Alone Less Frequently**  **n (%)** | **Used OAMs Alone Very Often**  **n (%)** | **Bivariate PR**  **(95% CI)** | **Adjusted PR**  **(95% CI)** |
| --- | --- | --- | --- | --- |
| **Total** | 66 (36.9)^b^ | 113 (63.1)^b^ | - | - |
| Age, Median (IQR) | 29 (25-40) | 31 (25-36) | 1.00 (0.98-1.01) | - |
| Female gender | 23 (34.8) | 37 (32.7) | 0.97 (0.76-1.23) | - |
| Race |  |  |  | - |
| *African American* | 8 (12.1) | 8 (7.1) | ref | - |
| *White* | 54 (81.1) | 90 (79.6) | 1.25 (0.75-2.07) | - |
| *Other* | 4 (6.1) | 15 (13.3) | 1.58 (0.92-2.72) | - |
| Temporary Housing in Past 3 Months | 33 (50.0) | 69 (61.1) | 1.18 (0.94-1.50) | - |
| Married or Living with Someone | 15 (22.7) | 17 (15.0) | 0.81 (0.58-1.15) | - |
| High School, GED, or Greater Education | 49 (74.2) | 90 (79.6) | 1.13 (0.84-1.51) | - |
| Symptoms of Major Depressive Disorder | 44 (66.7) | 66 (58.4) | 0.88 (0.71-1.10) | - |
| Used Heroin (& OAMs) ≥7 consecutive days^c^ | 50 (75.8) | 86 (76.1) | 1.01 (0.77-1.31) | - |
| Injected or Snorted any Drug Very Often^c^ | 43 (65.2) | 86 (76.1) | 1.23 (0.93-1.64) | - |
| Experienced an Overdose in Lifetime | 58 (87.9) | 106 (93.8) | 1.39 (0.80-2.41) | - |
| Experienced an Overdose in the Past Year | 45 (68.2) | 85 (75.2) | 1.14 (0.87-1.50) | - |
| Witnessed an Overdose in Lifetime | 58 (87.9) | 102 (90.3) | 1.10 (0.74-1.64) | - |
| Very Often Drank Alcohol within 2 hours of using OAMs^c^ | 4 (6.1) | 30 (26.5) | 1.54 (1.28-1.86) | 1.64 (1.33-2.03)^d^ |
| Very Often Took Sedatives within 2 Hours of Using OAMs^c^ | 2 (3.0) | 46 (40.7) | 1.87 (1.57-2.24) | 1.88 (1.57-2.25)^d^ |
| Very Often Used OAMs in an Unusual or New Place^c^ | 3 (4.5) | 72 (63.7) | 2.44 (1.91-3.10) | 2.48 (1.94-3.18)^d^ |

^a^Outcome modeled is those who used opioids Very Often vs. Often, Sometimes, Rarely, or Never. ^b^Percent of n=179 who used OAMs alone very often versus less frequently (often, sometimes, rarely, or never while alone). ^c^In the month before treatment or jail. ^d^Adjusted for age, race, gender, and injecting or snorting drugs very often in the month before treatment or jail. Abbreviations: IQR: interquartile range; OAMs: Opioid Analgesic Medications; PR: Prevalence Ratio; SD: standard deviation.

**Supplemental Table 3. Sensitivity Analysis: Overdose Risk Behaviors Associated with Using OAMs while Alone**

| **Associations of Overdose Risk Behaviors with Using OAMs while Alone “Often” or “Very Often”** | **Adjusted Prevalence Ratio**  **(95% CI)^a^** |
| --- | --- |
| Often or Very Often Drank Alcohol within 2 hours of Using OAMs (vs. Sometimes, Rarely, Never) | 1.13 (1.00-1.27) |
| Often or Very Often Took Sedatives within 2 Hours of Using OAMs (vs. Sometimes, Rarely, Never) | 1.30 (1.16-1.46) |
| Often or Very Often Used OAMs in an Unusual or New Place (vs. Sometimes, Rarely, Never) | 1.48 (1.24-1.76) |
| **Associations of Overdose Risk Behaviors with Using OAMs while Alone “Sometimes,” “Often,” or “Very Often”** | **Adjusted Prevalence Ratio**  **(95% CI)^b^** |
| Sometimes, Often, or Very Often Drank Alcohol within 2 hours of Using OAMs (vs. Rarely or Never) | 1.05 (0.98-1.13) |
| Sometimes, Often, or Very Often Took Sedatives within 2 Hours of Using OAMs (vs. Rarely or Never) | 1.06 (0.98-1.15) |
| Sometimes, Often, or Very Often Used OAMs in an Unusual or New Place (vs. Rarely or Never) | 1.18 (1.00-1.39) |

^a^Prevalence ratio adjusted for age, race, gender, and injecting or snorting drugs often or very often in the month before treatment or jail.

^b^Prevalence ratio adjusted for age, race, gender, and injecting or snorting drugs sometimes, often, or very often in the month before treatment or jail. Abbreviations: OAMs: Opioid Analgesic Medication

**Supplemental Table 4. Correlates of Using Heroin while Alone Very Often^a^**

| **Covariate** | **Used Heroin Alone Less Frequently**  **n (%)** | **Used Heroin Alone Very Often**  **n (%)** | **Bivariate PR**  **(95% CI)** | **Adjusted PR (95% CI)** |
| --- | --- | --- | --- | --- |
| **Total** | 59 (29.8)^b^ | 139 (70.2)^b^ | - | - |
| Age, Median (IQR) | 31 (27-39) | 28 (25-35) | 0.99 (0.98-1.00) | - |
| Female gender | 23 (39.0) | 49 (35.3) | 0.95 (0.79-1.16) | - |
| Race |  |  |  | - |
| *African American* | 6 (10.2) | 7 (5.0) | ref | - |
| *White* | 46 (78.0) | 111 (79.9) | 1.31 (0.79-2.19) | - |
| *Other* | 7 (11.9) | 21 (15.1) | 1.39 (0.81-2.41) | - |
| Temporary Housing in Past 3 Months | 31 (52.5) | 80 (57.6) | 1.06 (0.88-1.28) | - |
| Married or Living with Someone | 12 (20.3) | 19 (13.7) | 0.85 (0.63-1.15) | - |
| High School, GED, or Greater Education | 49 (83.1) | 111 (79.9) | 0.94 (0.76-1.17) | - |
| Symptoms of Major Depressive Disorder | 32 (54.2) | 85 (61.2) | 1.09 (0.90-1.32) | - |
| Used OAMs (& Heroin) ≥7 consecutive days^c^ | 36 (61.0) | 100 (71.9) | 1.17 (0.94-1.45) | - |
| Injected or Snorted any Drug Very Often^c^ | 30 (50.8) | 133 (95.7) | 4.76 (2.29-9.90) | - |
| Experienced an Overdose in Lifetime | 48 (81.4) | 132 (95.0) | 1.89 (1.05-3.39) | - |
| Experienced an Overdose in the Past Year | 32 (54.2) | 108 (77.7) | 1.44 (1.12-1.87) | - |
| Witnessed an Overdose in Lifetime | 55 (93.2) | 124 (89.2) | 0.88 (0.68-1.13) | - |
| Very Often Drank Alcohol within 2 hours of Using Heroin^c^ | 3 (5.1) | 13 (9.4) | 1.17 (0.91-1.51) | 1.17 (0.89-1.53)^d^ |
| Very Often Took Sedatives within 2 Hours of Using Heroin^c^ | 4 (6.8) | 37 (26.6) | 1.39 (1.19-1.62) | 1.17 (1.01-1.35)^d^ |
| Very Often Used Heroin in an Unusual or New Place^c^ | 4 (6.8) | 71 (51.1) | 1.71 (1.45-2.03) | 1.44 (1.21-1.70)^d^ |

^a^Outcome modeled is those who used opioids Very Often vs. Often, Sometimes, Rarely, or Never. ^b^Percent of total n=198 who used heroin alone very often versus less frequently (often, sometimes, rarely, or never while alone). ^c^In the month before treatment or jail. ^d^Adjusted for age, race, gender, injecting or snorting drugs very often in the month before treatment or jail, and ever personally experiencing an overdose. Abbreviations: IQR: interquartile range; OAMs: Opioid Analgesic Medications; PR: Prevalence Ratio; SD: standard deviation.

**Supplemental Table 5. Sensitivity Analysis: Correlates of Using Heroin while Alone**

| **Associations of Overdose Risk Behaviors with Using Heroin while Alone “Often,” or “Very Often”** | **Adjusted Prevalence Ratio**  **(95% CI)^a^** |
| --- | --- |
| Often or Very Often Drank Alcohol within 2 hours of Using Heroin (vs. Sometimes, Rarely, Never) | 1.06 (0.97-1.16) |
| Often or Very Often Took Sedatives within 2 Hours of Using Heroin (vs. Sometimes, Rarely, Never) | 1.02 (0.94-1.12) |
| Often or Very Often Used Heroin in an Unusual or New Place (vs. Sometimes, Rarely, Never) | 1.24 (1.09-1.41) |
| **Associations of Overdose Risk Behaviors with Using Heroin while Alone “Sometimes,” “Often,” or “Very Often”** | **Adjusted Prevalence Ratio**  **(95% CI)^b^** |
| Sometimes, Often, or Very Often Drank Alcohol within 2 hours of Using Heroin (vs. Rarely or Never) | 1.03 (0.97-1.09) |
| Sometimes, Often, or Very Often Took Sedatives within 2 Hours of Using Heroin (vs. Rarely or Never) | 1.01 (0.94-1.07) |
| Sometimes, Often, or Very Often Used Heroin in an Unusual or New Place (vs. Rarely or Never) | 1.22 (1.02-1.47) |

^a^Prevalence ratio adjusted for age, race, gender, injecting or snorting drugs often or very often in the month before treatment or jail, and ever personally experiencing an overdose.

^b^Prevalence ratio adjusted for age, race, gender, injecting or snorting drugs sometimes, often, or very often in the month before treatment or jail, and ever personally experiencing an overdose.

**Supplemental Table 6. Sensitivity Analysis: Behavioral and Psychosocial Correlates of Using any Opioid while Alone Often or Very Often^a^**

| **Covariate** | **Used Opioids Alone Less Frequently**  **n (%)** | **Used Opioids Alone Often or Very Often**  **n (%)** | **Bivariate PR (95% CI)** | **Adjusted PR (95% CI)** |
| --- | --- | --- | --- | --- |
|  |  |  |  |  |
| **Total** | 23 (9.5)^b^ | 218 (90.5)^b^ | - | - |
| Age, Median (IQR) | 34 (29-53) | 30 (25-36) | 0.99 (0.99-1.00) | 1.00 (0.99-1.00) |
| Female gender | 9 (39.1) | 74 (33.9) | 0.98 (0.89-1.07) | 0.98 (0.90-1.06) |
| Race |  |  |  |  |
| *African American* | 5 (21.7) | 14 (6.4) | ref | ref |
| *White* | 16 (69.6) | 174 (79.8) | 1.24 (0.95-1.63) | 1.11 (0.85-1.44) |
| *Other* | 2 (8.7) | 30 (13.8) | 1.27 (0.96-1.69) | 1.13 (0.87-1.48) |
| Temporary Housing in Past 3 Mos. | 11 (47.8) | 122 (56.0) | 1.03 (0.95-1.12) | - |
| Married or Living with Someone | 7 (30.4) | 36 (16.5) | 0.91 (0.79-1.05) | - |
| High School, GED, or Greater Education | 20 (87.0) | 175 (80.3) | 0.96 (0.88-1.05) | - |
| Symptoms of Major Depressive Disorder | 17 (73.9) | 125 (57.3) | 0.94 (0.87-1.01) | - |
| Heroin and OA Use, Mo. before Treat. or Jail |  |  |  |  |
| *Used OAs (No Heroin) ≥7 Consecutive Days* | 8 (34.8) | 35 (16.1) | ref | ref |
| *Used Heroin (No OAs) ≥7 Consecutive Days* | 7 (30.4) | 55 (25.2) | 1.09 (0.92-1.29) | 1.01 (0.84-1.21) |
| *Used Heroin & OAs ≥7 Consecutive Days* | 8 (34.8) | 128 (58.7) | 1.16 (1.00-1.34) | 1.05 (0.88-1.24) |
| Injected or Snorted any Drug Often or Very Often, Mo. before Treat. or Jail | 13 (56.5) | 191 (87.6) | 1.28 (1.05-1.57) | 1.17 (0.95-1.45) |
| Experienced an Overdose in Lifetime | 17 (73.9) | 201 (92.2) | 1.25 (0.98-1.60) | 1.19 (0.97-1.45) |
| Experienced an Overdose in the Past Year | 12 (52.2) | 154 (70.6) | 1.09 (0.98-1.21) | - |
| Witnessed an Overdose in Lifetime | 22 (95.7) | 194 (89.0) | 0.94 (0.85-1.03) | - |
| Self-Stigma (Self-Devaluation Z-Score), Mean (SD) | -0.05 (1.07) | 0.00 (0.99) | 1.00 (0.96-1.05) | - |
| Self-Stigma (Self-Devaluation Score Top Quartile)^c^ | 6 (26.1) | 45 (20.6) | 0.97 (0.87-1.08) | 0.91 (0.80-1.03) |
| Anticipated Stigma (Fear of Enacted Stigma Z-Score), Mean (SD) | -0.27 (1.17) | 0.03 (0.98) | 1.03 (0.98-1.08) | - |
| Anticipated Stigma (Fear of Enacted Stigma Score Top Quartile)^d^ | 3 (13.0) | 55 (25.2) | 1.06 (0.98-1.15) | 1.06 (0.97-1.17) |

^a^Outcome modeled is those who used opioids Very Often or Often vs. Sometimes, Rarely, or Never. ^b^Percents among total n=241. ^c^Top quartile: ≥35 points. ^d^Top quartile: ≥39 points. Abbreviations & Acronyms: IQR: Interquartile Range; Mo: Month; OAM: Opioid Analgesic Medication; PR: Prevalence Ratio; SD: Standard Deviation; Treat: Treatment.
